# Soybean Flour Fortified with *Gryllus assimilis* Powder to Increase Iron Bioavailability Improves Gut Health and Oxidative Balance In Vivo

**DOI:** 10.3390/nu17030437

**Published:** 2025-01-25

**Authors:** Michele Lílian da Fonseca Barnabé, Laura Célia de Oliveira Souza Vicente, Karina Vitoria Cipriana Martins, Gabrieli Fernandes Lacerda, Elias Rodrigues, Lívya Alves Oliveira, Kelly Aparecida Dias, Stephanie Michelin Santana Pereira, Vinicius Parzanini Brilhante de São José, Manoela Maciel dos Santos Dias, Ricardo C. Calhelha, Luciano Bernardes Leite, Lúcia Ribeiro, Izabela Maria Montezano de Carvalho, Bárbara Pereira da Silva, Hércia Stampini Duarte Martino, Reggiani Vilela Gonçalves, Ceres Mattos Della Lucia

**Affiliations:** 1 Laboratory of Vitamin Analysis, Department of Nutrition and Health, Universidade Federal de Viçosa, Viçosa 36570-900, MG, Brazil; michele.barnabe@ufv.br (M.L.d.F.B.); lauraceliao@gmail.com (L.C.d.O.S.V.); karina.cipriana@ufv.br (K.V.C.M.); gabrieli.lacerda@ufv.br (G.F.L.); elias.rodrigues1@ufv.br (E.R.); livya.oliveira@ufv.br (L.A.O.); kelly.dias@ufv.br (K.A.D.); stephanie.pereira@ufv.br (S.M.S.P.); cmdellalucia@ufv.br (C.M.D.L.); 2 Laboratory of Experimental Nutrition, Department of Nutrition and Health, Universidade Federal de Viçosa, Viçosa 36570-900, MG, Brazil; vinicius.sao@ufv.br (V.P.B.d.S.J.); barbara.p.silva@ufv.br (B.P.d.S.); hercia@ufv.br (H.S.D.M.); 3 Department of Animal Biology, Universidade Federal de Viçosa, Viçosa 36570-900, MG, Brazil; manoelamaciel80@gmail.com (M.M.d.S.D.); reggiani.goncalves@ufv.br (R.V.G.); 4 Centro de Investigação de Montanha (CIMO), Instituto Politécnico de Bragança, Alameda Santa Apolónia, 5300-252 Bragança, Portugal; lucia.maiaribeiro@gmail.com; 5 Laboratório Associado para a Sustentabilidade e Tecnologia em Regiões de Montanha (SusTEC), Instituto Politécnico de Bragança, Campus de Santa Apolónia, 5300-252 Bragança, Portugal; 6 Department of Physical Education, Universidade Federal de Viçosa, Viçosa 36570-900, MG, Brazil; luciano.leite@ufv.br; 7 Department of Sports, Instituto Politécnico de Bragança, 5300-252 Bragança, Portugal; 8 Facultade de Ciencias, Universidad de Vigo, 32004 Ourense, Spain; 9 Laboratory of Food Analysis, Department of Nutrition and Health, Universidade Federal de Viçosa, Viçosa 36570-900, MG, Brazil; zabela.carvalho@ufv.br

**Keywords:** minerals, biological availability, entomophagy, edible insects

## Abstract

Background: Insects like *Gryllus assimilis* have an excellent nutritional profile, including iron. However, the bioavailability of this iron and its effects on intestinal health and oxidative balance remain unclear. To enhance acceptance, insects can be used in powder form and combined with common flours. Objective: This study evaluates the effects of *Gryllus assimilis* powder, alone or with soy flour, on iron bioavailability, intestinal health, and oxidative balance in rodents. Methods: Using the hemoglobin depletion/repletion method, 32 male Wistar rats were divided into four groups: A (standard diet + ferrous sulfate), B (diet + *Gryllus assimilis* + soy flour), C (diet + *Gryllus assimilis*), and D (diet + soy flour). Hemoglobin levels, regeneration efficiency, biological value, serum markers, intestinal health, and oxidative balance were assessed. Results: Food intake, weight gain, and bioavailability measures showed no differences. However, the Gryllus + soy group showed higher weekly and final hemoglobin levels than Gryllus alone. This combination also improved acetic acid levels, fecal moisture, and oxidative balance, increasing superoxide dismutase activity while reducing peroxidation products compared to Gryllus alone. Conclusion: These findings highlight the potential benefits of combining *Gryllus assimilis* with soy flour for iron bioavailability and overall health.

## 1. Introduction

Iron is an essential mineral for the body, playing crucial roles in oxygen transport and cellular metabolism. Its deficiency mainly occurs when the dietary intake is insufficient or when there is an imbalance between intake and absorption, resulting in conditions such as anemia [1,2,3]. Iron deficiency anemia, in particular, affects more than 160,000 preschool and school-aged children in Latin America and the Caribbean, highlighting the importance of ensuring adequate dietary sources of iron [4].

An important factor in the effectiveness of dietary iron sources is bioavailability, which refers to the fraction of the ingested nutrient that becomes available for use and storage in the body [5]. Iron bioavailability is influenced by physiological and dietary factors [6,7], and, in recent years, the potential of alternative nutrient sources, such as edible insects, has been studied. These sources, in addition to being rich in iron, may offer nutritional and environmental advantages [8,9,10].

*Gryllus assimilis*, an edible cricket native to Latin America, has garnered interest as one of these alternative sources. This species can be successfully reared in controlled environments and is a rich source of essential minerals, such as copper, manganese, zinc, and iron (2.78 mg/100 g) [11,12]. Additionally, due to its content of fiber, vitamins, and antioxidant compounds, the consumption of *Gryllus assimilis* may offer gastrointestinal health benefits and support antioxidant and anti-inflammatory functions [13,14,15,16,17,18]. Similarly, soy (*Glycine max* (L.) Merrill), widely cultivated in Brazil, is a legume rich in high-quality proteins, providing an excellent source of essential amino acids such as lysine and tryptophan, both of which are limited in some protein sources, including insect powders [19,20]. Thus, the combination of cricket and soy flours may represent an interesting nutritional strategy to complement amino acid profiles, enhancing protein quality [21].

Insect flour has emerged as a functional and sustainable alternative, rich in bioactive compounds that enhance iron absorption and may benefit intestinal health. Peptides derived from its proteins and some amino acids act as natural chelators, increasing iron solubility and bioavailability. Thus, the enhancing effect of protein on iron absorption may be explained by sulfur-containing amino acids that keep iron in solution and available for absorption in the digestive tract [22]. In addition, components such as chitosan and chitin can modulate the intestinal microbiota, creating a favorable environment for nutrient absorption. Few studies have explored insect consumption through in vivo or in vitro studies on iron bioavailability [23,24]. Thus, our study presents an innovative character and can support future studies.

The consumption of antioxidant-rich foods is also essential, as oxidative stress, resulting from an imbalance between reactive oxygen species (ROS) and the body’s antioxidant system, is associated with the impairment of intestinal barrier integrity, increased permeability, and the onset of intestinal diseases [25,26]. This oxidative stress can trigger inflammation and affect the intestinal absorption of nutrients, such as iron, by interfering with the regulation of mineral transport proteins [27,28].

Soy isoflavones play a significant role in neutralizing ROS. Their antioxidant mechanism is mainly based on the donation of electrons by phenolic groups, resulting in the neutralization of free radicals. In addition, their antioxidant properties are also associated with the regulation of the activity of antioxidant enzymes, such as superoxide dismutase (SOD), catalase (CAT), and glutathione peroxidase (GPx), through the activation of the Nrf2 pathway [29,30].

Thus, the combination of *Gryllus assimilis* flour with soy can enhance the functional benefits by combining the high protein and bioavailable iron content of insects with the antioxidant and gut-health-modulating effects of soy. The literature lacks studies on the bioavailability of iron in *Gryllus assimilis* powder and the effects of its consumption, whether alone or in combination with soy flour, on intestinal health and oxidative balance in vivo.

Our study aims to fill this gap by investigating the absorption and efficacy of iron from these edible insects, as well as evaluating potential impacts on the gut health and antioxidant system. Understanding these aspects is essential to support insect consumption by the population, foster the development of new food products, and identify alternative nutrient sources with the potential to improve nutritional profiles in developing regions.

## 2. Materials and Methods

### 2.1. Obtaining and Preparing Flours

Black cricket powder (*Gryllus assimilis*) was obtained from Ecological Food, a company located in Limeira, São Paulo, specializing in the rearing of insects for animal feed. These insects were raised on a diet composed of wheat bran, quail feed (free of meat or animal by-products), carrot, and water. They were kept in a controlled breeding environment with a temperature maintained between 28 °C and 30 °C. Upon reaching adulthood, the crickets were euthanized by freezing, dehydrated, and processed into powder form.

The soybeans (UFVTN 105 AP variety) used in the study were provided by the Soybean Breeding Program at the Biotechnology Institute of the Federal University of Viçosa (BIOAGRO/UFV), developed through a backcrossing method. To produce the flour, the soybeans were ground using a knife mill equipped with a 600 µm sieve (30 mesh size) and a vertical rotor grinder (MA 090 CFT, Marconi Equipment, Piracicaba, SP, Brazil), resulting in a final particle size of 850 µm. The soybean flour was then packed in polyethylene bags and stored in a freezer at −18 °C until analysis.

### 2.2. Chemical Composition of Soy Flour and Cricket Powder

The proximate composition analysis of the flours was conducted according to methods described by the AOAC [31]. The moisture content was determined by gravimetry, and the fiber content by enzymatic gravimetric technique. The lipid content was measured using the Soxhlet method, while ash was determined by incineration in a muffle furnace and the protein content by the Kjeldahl method. Carbohydrates were calculated by difference, and the iron content was quantified by atomic absorption spectrometry. All analyses were performed in triplicates. The chemical composition values obtained were used to calculate the experimental diets, ensuring they were isocaloric and contained the same macronutrient levels per 100 g.

### 2.3. Iron Determination

The iron content in *Gryllus assimilis* and soy flour, as well as in the experimental diets and albumin, was determined by atomic absorption spectrometry (SpectrAA, Model 220 FS, Varian, Inc., Palo Alto, CA, USA). Analyses were conducted under conditions of a 1300 W power, an argon plasma flow rate of 15 L min⁻^1^, an auxiliary argon flow rate of 0.7 L min⁻^1^, an argon nebulizer flow rate of 0.5 L min⁻^1^, and a sample introduction rate of 1.5 mL min⁻^1^. Separate 2 g samples of each (cricket powder and soy flour) were weighed on parchment paper using an analytical balance (Gehaka, BG2000, São Paulo, SP, Brazil) and transferred to digestion tubes. Next, 10 mL of nitric acid at room temperature was added to the tubes, which were then held in a digestion block at 150 °C for 16 h. When necessary, an additional 5 mL of nitric acid was added to the tubes. After digestion, the tubes were cooled to room temperature, and the samples were transferred to 50 mL volumetric flasks. The tubes were rinsed with deionized water and vortexed three times. For analysis, the extracts were appropriately diluted with deionized water. All analyses were performed in triplicates.

### 2.4. Experimental Animals and Diets

The method used to analyze iron bioavailability was the hemoglobin depletion/repletion technique. Thirty-two young male Wistar rats (*Rattus norvegicus*, albinus variety), 21 days old, recently weaned, from the Central Animal Facility of the Center for Biological and Health Sciences, Federal University of Viçosa, with an average initial weight of 60 g, were used. The sample calculation was performed as proposed by Fontelles et al. [32]. They remained in a temperature-controlled environment (21 ± 1 °C), in individual stainless steel cages (18 cm high × 20 cm wide × 31 cm long), under a 12 h light–dark cycle automatically controlled. The cages were previously demineralized using a 10% nitric acid (HNO_3_) solution for 24 h and were subsequently rinsed with deionized water. At the end of the depletion phase, the animals weighed between 126 and 171 g, while, at the end of the repletion phase, their weight ranged from 137 to 210 g.

The experimental diets were prepared following the AIN-93G standard. During the depletion phase, the animals were given a diet containing an iron-free mineral mix to reduce hemoglobin levels, with ad libitum access to deionized water for 21 days. Subsequently, the animals were divided into four groups (n = 8) to ensure homogeneous hemoglobin levels in each group: (1) standard diet + ferrous sulfate (SD + FS), (2) standard diet + cricket powder (15%) + soy flour (85%) (SD + CP + SF), (3) standard diet + cricket powder (100%) (SD + CP), (4) standard diet + soy flour (100%) (SD + SF) (Table 1).

During the repletion phase, both *Gryllus assimilis* powder and soy flour were used as sources of iron, with ferrous sulfate serving as the positive control. The 15:85 ratio of *Gryllus assimilis* powder to soy flour in the experimental diets was established to meet the required iron content of 12 mg/kg of diet during the repletion phase. This proportion was determined through calculations ensuring the dietary iron levels were sufficient to promote hemoglobin repletion, while also balancing the nutritional contributions of both ingredients. Throughout the repletion phase, blood samples were collected weekly via a tail puncture to determine the hemoglobin concentration. After 48 days of experimentation and a 12 h fast, the animals were anesthetized with isoflurane (Isoforine^®^ Cristália, Itapira, SP, Brazil) and euthanized by cardiac puncture.

Blood was collected in test tubes and centrifuged at 3000 rpm for 10 min (FANEM^®^, São Paulo, Brazil) to separate the serum. Organs such as the liver and intestine were also collected. All biological materials were stored at −80 °C for further analysis.

All procedures followed ethical principles of animal experimentation. The study was approved by the Animal Use Ethics Committee of the Federal University of Viçosa (CEUA/UFV), under registration 38/2022, 16 December 2022.

### 2.5. Hemoglobin and Serum Ferritin and Transferrin Assays

Serum hemoglobin was measured using the cyanmethemoglobin method proposed by AOAC [33], employing an in vitro colorimetric diagnostic kit. A volume of 20 μL of blood was pipetted and mixed with 5 mL of Drabkin’s reagent solution (containing potassium cyanide and hydrocyanic acid). Absorbance readings were taken using a Multiskan UV-visible spectrophotometer (Thermo Fisher Scientific^®^, Waltham, MA, USA) at a wavelength of 540 nm. The hemoglobin concentration in the blood samples was calculated by referencing the absorbance reading against a standard hemoglobin solution.

### 2.6. Iron Bioavailability

Iron bioavailability was determined according to the method proposed by López-Alarcón et al. [34]. Hemoglobin regeneration efficiency (HRE%) was calculated using the following formula: HRE% = [(final Hb Fe mg − initial Hb Fe mg)/100]/mg Fe consumed. The iron content in the hemoglobin was estimated using: [body weight (g) × Hb (g L⁻^1^) × 0.335 × 6.7]/1000. This variable was calculated assuming that the total blood volume equals 6.7% of the body weight and that the iron content of the hemoglobin in the body is 0.335. Iron utilization was calculated as [HRE% × % dietary iron]/100. The relative biological value was determined by the ratio [(HRE% of the test group)/(HRE% of the control group)].

### 2.7. Oxidative Stress Biomarkers

#### 2.7.1. Tissue Preparation

Frozen liver tissue samples (100 mg) were homogenized (using the Tissue Master 125 homogenizer, OMNI International, Kennesaw, GA, USA) in 1 mL of phosphate buffer (pH 7.4), centrifuged for 10 min at 10,000× *g* (12,000 rpm), and kept under refrigeration at 4 °C. The resulting supernatants were collected for analysis of the enzymatic activity of superoxide dismutase (SOD), catalase (CAT), glutathione S-transferase (GST), malondialdehyde content (MDA), nitric oxide (NO), and total protein (TP) assays. The resulting pellets were used for the analysis of carbonyl protein (CP) content. These analyses were performed in an ELISA microplate reader (Multiskan GO, Thermo Fisher Scientific^®^, Waltham, MA, USA) or a spectrophotometer (UV-Mini 1240, Shimadzu, Kyoto, Japan).

#### 2.7.2. Catalase Activity

The determination of catalase (CAT) enzyme activity was based on its ability to cleave hydrogen peroxide (H_2_O_2_) into water and molecular oxygen, as described by Aebi [35]. Absorbance measurements were recorded at 0, 30, and 60 s at 240 nm using a UV/VIS spectrophotometer (T70+ Spectrometer, PG Instruments, Leicestershire, United Kingdom). For calculation purposes, the absorbance used was the delta value derived from the difference between the final (60 s) and the initial (0 s) absorbance readings.

#### 2.7.3. Superoxide Dismutase Activity

The quantification of superoxide dismutase (SOD) was performed according to the methodology proposed by Marklund [36]. The samples, standard, and blank were incubated at 37 °C for 5 min, and the absorbance was measured at 570 nm using a Multiskan GO spectrophotometer (Thermo Fisher Scientific^®^, Waltham, MA, USA). Results are expressed as units of SOD per mg of protein. Calculations were based on the absorbance value of the standard, which was considered to have 1 U of SOD, corresponding to a 100% oxidation of pyrogallol.

#### 2.7.4. Glutathione S-Transferase Activity

The activity of glutathione-S-transferase (GST) was determined according to Habig et al. [37]. After centrifuging the homogenate, CDNB (Sigma Aldrich^®^, St. Louis, MO, USA) and reduced glutathione (GSH) (Sigma Aldrich^®^, St. Louis, MO, USA) were added. Absorbance readings were taken at 0, 30, 60, and 90 s at 340 nm using a spectrophotometer (Multiskan GO, Thermo Fisher Scientific^®^, Waltham, MA, USA).

#### 2.7.5. Malondialdehyde Concentration

The concentration of malondialdehyde (MDA) was measured according to Buege and Aust [38]. A TBARs solution (containing 15% trichloroacetic acid, 0.375% thiobarbituric acid, and 0.25 M HCl) was added to the liver homogenate. The samples were then vortexed and heated in a water bath at 95 °C for 30 min. Following this procedure, *n*-butanol was added, followed by further vortexing and centrifugation. The supernatant was removed and pipetted in triplicates onto an Elisa plate for spectrophotometric readings at 535 nm using a Multiskan GO (Thermo Fisher Scientific^®^, Waltham, MA, USA). Results are expressed as nmol of MDA per milligram of protein (MDA/PROT).

#### 2.7.6. Nitric Oxide Concentration

The concentration of nitric oxide (NO) was determined according to the method proposed by Green et al. [39]. The homogenate was mixed with two solutions: solution A (1% sulfanilamide in 2.5% H_3_PO_4_) and solution B (0.1% *N*-(1-naphthyl) ethylenediamine dihydrochloride in 2.5% H_3_PO_4_) in a 1:1 ratio. The microtiter plate was then incubated in the dark for 10 min. Absorbance was measured at 570 nm using a spectrophotometer (Multiskan Go, Thermo Scientific), and the results were calculated using a standard curve and are expressed in µmol NO/mg of protein.

#### 2.7.7. Carbonylated Protein Concentration

The concentration of carbonylated protein was measured using the 2,4-dinitrophenylhydrazine (DNPH) procedure based on the reaction of carbonyl groups with DNPH, as described by Levine et al. [40]. Absorbance was measured at 370 nm using a spectrophotometer (Multiskan GO, Thermo Fisher Scientific^®^, Waltham, MA, USA).

#### 2.7.8. Total Protein Concentration

The determination of total protein content was carried out following the method outlined by Lowry et al. in 1951 [41], utilizing bovine serum albumin as the standard. The total protein concentrations obtained were then used to standardize the results for CAT, SOD, MDA, and PC.

### 2.8. Analysis of Fecal Content and Duodenal Morphology

#### 2.8.1. Fecal Moisture

The moisture content of feces was determined by the gravimetric method according to Paez et al. [42]. Samples collected at the end of the experiment were dried in an oven at 105 °C for 24 h.

#### 2.8.2. Fecal Color and Consistency (Bristol Stool Scale)

To gather information on intestinal transit and function, the Bristol Stool Scale for Stool Consistency (BSS) was used. A fecal color analysis was conducted through macroscopic observation, adapting the classification proposed by Silveira Júnior [43], while stool consistency was assessed using a scoring system previously proposed by Canani et al. [44].

#### 2.8.3. Cecal Fecal pH Analysis

For the pH analysis, approximately 0.4 g of cecal content was homogenized in 4 mL of distilled water using a vortex mixer (Vortex LP, Thermo Fisher Scientific^®^, Waltham, MA, USA). A glass electrode pH meter (Bel Engineering^®^, Monza, Italy) was then used to measure fecal pH [45].

#### 2.8.4. Quantification of Short-Chain Fatty Acids (SCFAs)

For short-chain fatty acid quantification, the method proposed by Siegfried et al. [46] was followed. Cecal feces were mixed in an Eppendorf tube with 300 μL of ultrapure water in microtubes and centrifuged at 12,000× *g* for 10 min. Then, 300 μL of the supernatant was transferred to new tubes, to which 300 μL of calcium hydroxide and 150 μL of copper sulfate solution was added. The tubes were shaken for 10 s and then frozen. After thawing, they were centrifuged again at 12,000× *g* for 10 min. Five hundred microliters of the supernatant was transferred to new microtubes, 14 μL of concentrated H_2_SO_4_ was added, and the tubes were frozen once more. After thawing and centrifuging, 500 μL of the supernatant was used for SCFA quantification.

#### 2.8.5. Histological Analysis of the Duodenum

Semi-serial histological fragments of the colon and duodenum, 3 μm thick, were obtained using an automated rotary microtome (Reichert-Jung^®^, Genossen, Germany) and stained using the hematoxylin and eosin technique. The slides were examined under an Olympus BX43 light microscope with 20× and 40× objectives. To measure the thickness of the mucosal and submucosal layers and the diameter of goblet cells, 10 random fields per animal were selected. Only crypts with a defined and visible connective epithelium were used. The images were processed with the ImagePro-Plus^®^ software, version 4.5 (Media Cybernetics, Rockville, MD, USA).

### 2.9. Statistical Analysis

Data were subjected to Shapiro–Wilk and Kolmogorov–Smirnov normality tests. A one-way analysis of variance (ANOVA) was applied, followed by a Tukey’s post-hoc test for parametric variables. The significance level for all tests was set to 5%. All analyses were performed using the GraphPad Prism software (GraphPad Software, San Diego, CA, USA), version 9.0. A 5% significance level was established for all tests.

## 3. Results

The proximate composition analysis revealed that the *Gryllus assimilis* powder contained 23.72 mg of iron per 100 g, while the soy flour had 7.94 mg of iron per 100 g (Table 1).

No differences in food intake or weight gain were observed among the animals in the different groups (*p* > 0.05) (Table 2). However, animals that consumed *Gryllus assimilis* powder combined with soy flour (GP + FS) had higher iron intake compared to the group that consumed soy flour alone (FS) (*p* < 0.05) (Table 2). The iron utilization rate was higher in the GP + FS group compared to the group that consumed *Gryllus assimilis* powder alone (*p* < 0.05) (Table 2). No differences were found in Hemoglobin Regeneration Efficiency (HRE%), Relative Bioavailability Value (RBV-HRE), or Feed Efficiency Ratio (FER) among the groups (*p* > 0.05) (Table 2).

At the beginning of the repletion phase, hemoglobin levels did not differ between the test and control groups (*p* > 0.05) (Table 3). However, in the final week, the GP group showed lower hemoglobin levels compared to the other groups (*p* < 0.05), with a final hemoglobin gain smaller than that of the other groups (*p* < 0.05) (Table 3). The groups that consumed *Gryllus assimilis* powder alone (GP) or combined with soy flour (GP + FS) exhibited higher transferrin levels at the end of the study (*p* < 0.05) compared to the SF and FS groups. Ferritin levels, on the other hand, were higher in the GP group (*p* < 0.05) compared to the GP + FS group (Table 3).

The group that received soy flour alone (FS) showed a higher concentration of acetic acid in the cecal content (*p* < 0.05) compared to the other groups. Propionic acid concentrations were also higher in the FS group (*p* < 0.05) compared to the SF and GP + FS groups. Butyric acid concentrations did not differ among the groups (*p* > 0.05) (Table 4). Fecal moisture was higher (*p* < 0.05) in the GP + FS group compared to the SF and GP groups. No differences were observed in cecal fecal pH or color among the groups (*p* > 0.05) (Table 4).

**Figure 1:**
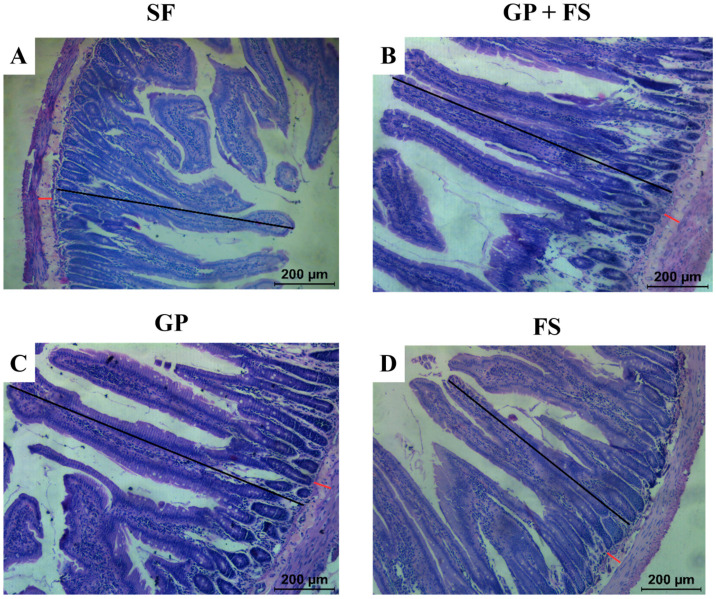
Histological section of the intestinal epithelium (duodenum) for the analysis of the mucosa and submucosa. Cross section of the duodenum of Wistar rats subjected to an analysis of iron bioavailability using the depletion/repletion method. (**A**) SF: Ferrous Sulfate group; (**B**) GP + FS: *Gryllus assimilis* powder associated with soy flour (15:85) group; (**C**) GP: *Gryllus assimilis* Powder group; (**D**) FS: Soy Flour group. The black lines indicate how the measurements of the mucosal thickness were made. The red lines indicate how the measurements of the submucosal thickness were made.

**Figure 2:**
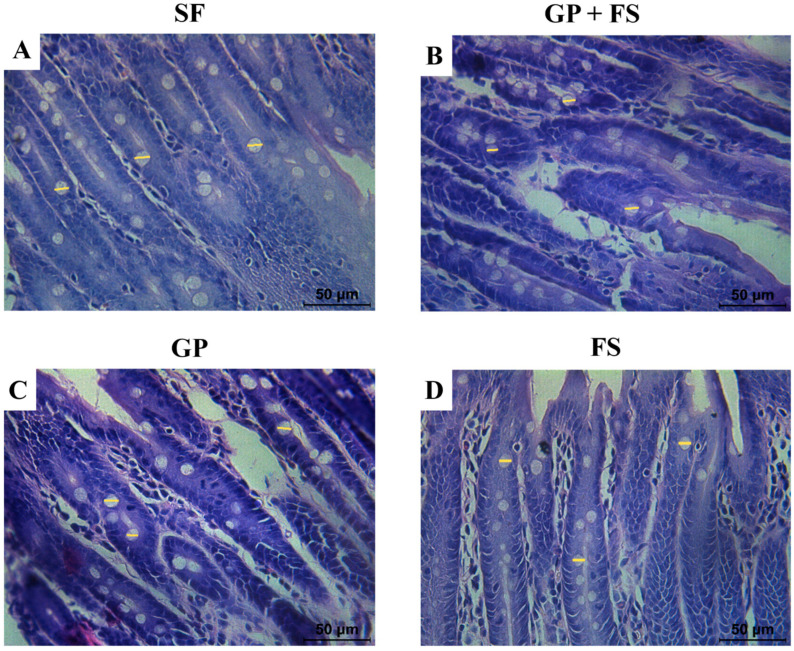
Histological section of the intestinal epithelium (duodenum) for the analysis of the goblet cells. Cross section of the duodenum of Wistar rats subjected to an analysis of iron bioavailability using the depletion/repletion method. (**A**) SF: Ferrous Sulfate group; (**B**) GP + FS: *Gryllus assimilis* powder associated with soy flour (15:85) group; (**C**) GP: *Gryllus assimilis* Powder group; (**D**) FS: Soy Flour group. The yellow lines indicate how the measurements of the diameters of the goblet cells were made.

A histomorphometric analysis of the duodenum showed that the GP and FS groups had a greater mucosal thickness compared to the SF group (Table 5, Figure 1 and Figure 2). However, submucosal thickness did not differ among the groups (*p* > 0.05). The consumption of *Gryllus assimilis* powder alone promoted a larger diameter of goblet cells (*p* < 0.05) (Table 5, Figure 1 and Figure 2).

**Figure 3:**
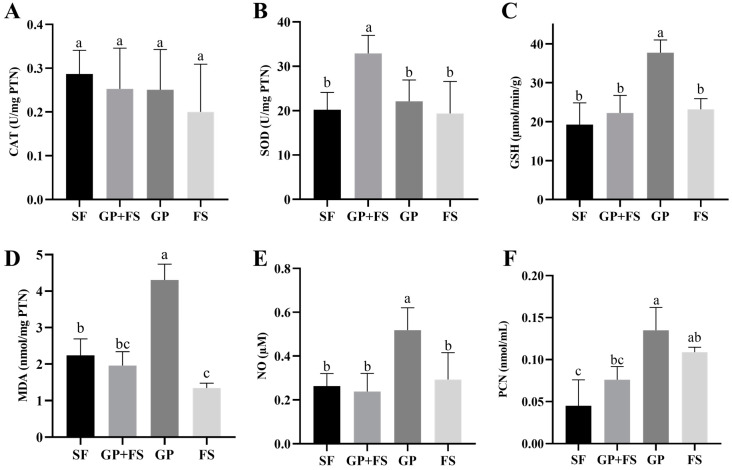
Effect of consumption of *Gryllus assimilis* powder alone or associated with soy flour on in vivo oxidative balance. SF: Ferrous Sulfate; GP + FS: *Gryllus assimilis* powder associated with soy flour (15:85); GP: *Gryllus assimilis* Powder; FS: Soy Flour. (**A**) Catalase, (**B**) superoxide dismutase, (**C**) glutathione S-transferase, (**D**) malondialdehyde, (**E**) nitric oxide, and (**F**) protein carbonylate. Different lowercase letters (a–c) indicate intragroup differences, according to ANOVA tests followed by a Tukey’s test at a 5% probability. Data are expressed as the mean ± standard deviation.

Antioxidant activity and oxidative stress were also analyzed in the liver. CAT values showed no differences among groups (*p* > 0.05). However, SOD concentrations were higher in the GP + SF group (*p* < 0.05), while GSH concentrations were higher in the GP group (*p* < 0.05) (Figure 3). Conversely, the production of NO, MDA, and PCN was higher in the group that received *Gryllus assimilis* powder alone (GP) (*p* < 0.05) (Figure 3).

## 4. Discussion

In the present study, the effect of consuming *Gryllus assimilis* powder, alone or in combination with soy flour, on iron bioavailability, intestinal health, and oxidative balance was evaluated in rodents. The main findings show that the combination of *Gryllus assimilis* powder with soy flour effectively maintained weekly hemoglobin levels and attained a final hemoglobin gain compared to *Gryllus assimilis* consumption alone. Additionally, this combination resulted in improvements in intestinal health, with a higher concentration of acetic acid and increased fecal moisture compared to soy flour alone. A positive effect on oxidative balance was also observed, evidenced by increased superoxide dismutase activity, while the group consuming *Gryllus assimilis* alone showed higher concentrations of peroxidation products.

Initially, no differences in food intake and weight gain were observed among the groups, indicating good acceptance of the offered diets. Similar findings have been reported in other studies with *Gryllus assimilis* powder [21,47,48]. Additionally, the combination of *Gryllus assimilis* with soy flour exhibited iron bioavailability levels comparable to those of ferrous sulfate, as indicated by the HRE% and RBV-HRE values which did not differ among the groups. The absence of significant differences revealed by these outcomes between the experimental groups and the ferrous sulfate group—considered the gold standard for iron supplementation—underscores the potential efficacy of the *Gryllus assimilis* and soy flour combination. These null findings are, in fact, positive, as they suggest that the experimental diet performs comparably to ferrous sulfate in terms of supporting hemoglobin regeneration and iron bioavailability. This highlights the promising potential of *Gryllus assimilis* as a viable alternative source of iron.

However, the consumption of *Gryllus assimilis* alone resulted in lower hemoglobin concentrations in the final week, a lower final hemoglobin gain, and a reduced iron utilization rate. These findings suggest that, although *Gryllus assimilis* is effective for hemoglobin regeneration, its isolated consumption may hinder the maintenance of hemoglobin levels over the long term. Additionally, the groups consuming cricket powder, either alone or combined with soy, showed higher serum transferrin concentrations compared to the control group.

Studies suggest that, although edible insects, such as *Gryllus assimilis*, are sources of bioavailable iron, compounds present in their exoskeleton, such as chitin, may interfere with mineral absorption. Chitin, an abundant polymer in exoskeletons, can act as a chelator, reducing the absorption of essential minerals, including iron, calcium, and zinc [9,20,49,50,51]. This effect may help explain the lower hemoglobin concentrations observed in the group that consumed *Gryllus assimilis* alone. Furthermore, the increase in transferrin observed in the GP group may be associated with iron metabolism, as a large portion of plasma iron originates from hemoglobin degradation and body stores, such as ferritin [52,53]. This process generates a greater demand for iron transport, elevating plasma transferrin levels [54,55], a phenomenon similar to findings by Abdel-Monien et al. [56] in iron-deficient rats supplemented with iron. Although ferritin concentrations did not differ significantly between the intervention and control groups, the GP-only group showed higher ferritin levels compared to the group combined with soy, a phenomenon which may reflect a mild inflammatory response. In inflammatory conditions, ferritin commonly increases, leading to reduced iron availability for metabolic processes [57,58]. Therefore, the isolated consumption of *Gryllus assimilis* may have triggered a mild inflammatory response in the animals.

Improvements in intestinal parameters were observed in the test groups, especially with the inclusion of soy flour. Although butyric acid concentrations did not differ among groups, the SF group showed higher levels of acetic and propionic acids, followed by the GP group, a phenomenon which may be attributed to phytochemicals in soy, such as isoflavones, phytosterols, and saponins, known to promote gastrointestinal health benefits [59]. Isoflavones, in particular, enhance intestinal barrier integrity and have anti-inflammatory effects [60]. Soy also appears to increase short-chain fatty acids (SCFAs) through microbial fermentation, as shown by Huang et al. [61] and Li et al. [62], promoting beneficial effects on barrier function and reducing intestinal inflammation [63,64].

Additionally, the higher fecal moisture in the groups that received soy and cricket powder may be related to insoluble fiber content, which increases fecal bulk and accelerates intestinal transit [65]. Fibers also help with weight management and the regulation of metabolic diseases, such as type 2 diabetes [66,67]. These intestinal improvements were accompanied by histomorphometric changes, with a greater mucosal thickness in the test groups and an increase in goblet cell diameter in the GP-only group, suggesting an adaptation of the digestive tract to optimize nutrient absorption [68]. When degraded, the chitin in insect exoskeletons produces chitooligosaccharides (COS), which play a role in immune modulation and support intestinal health [9,69]. Such changes may even enhance iron absorption, as mucus facilitates its uptake [52,70].

In the present study, animals that received *Gryllus assimilis* powder alone showed a higher expression of oxidative stress biomarkers and increased glutathione S-transferase (GST) activity. GST is known to be the central enzyme in the reaction with glutathione (GSH), a non-enzymatic antioxidant that acts as a primary detoxifying agent by eliminating accumulated foreign materials from the body and playing a crucial role in protecting cells against damage caused by free radicals [71,72]. Thus, it is hypothesized that *Gryllus assimilis* powder, when consumed alone, exerted an oxidizing effect on the animals, and the increased GST expression occurred adaptively to minimize the oxidative damage induced by this stress. Under these conditions, the endogenous antioxidant system, including glutathione, can be induced as a compensatory mechanism. However, when the production of reactive oxygen species (ROS) exceeds an antioxidant capacity, biomolecules such as lipids can suffer oxidative damage, reflected in elevated MDA levels. Although contradictory, the results demonstrate the complexity of the redox balance in the organism, since increased antioxidant responses are not always sufficient to completely mitigate the oxidative damage caused, for example, during hemoglobin depletion.

The SOD is an enzyme capable of converting harmful superoxide radicals (O_2_⁻) into hydrogen peroxide (H_2_O_2_) and oxygen (O_2_). When *Gryllus assimilis* powder was supplemented with soy flour, lipid peroxidation product levels were similar to the control, and SOD activity increased. The antioxidant capacity of diets containing edible insects has also been studied by Xie et al. [73], who found that powdered *Chrysomyia megacephala* larvae increased SOD expression in mice. The increase in this antioxidant enzyme activity in the GP + SF group in the present study may be associated with the potentiation of bioactive compounds present in crickets [16]. Soy contains isoflavones and saponins, while crickets contain glycosaminoglycans and peptide fractions. Together, these food matrices contain bioactive compounds with antioxidant and antimicrobial properties that can reduce inflammation and oxidative stress [14,15,74,75].

There are reports that cricket powder consumption results in anti-inflammatory and antioxidant activity in rodents [76,77]. Contrarily, greater oxidative activity was observed in animals that consumed this food matrix alone, as evidenced by the increased expression of peroxidation products. Higher MDA and PCN expression is associated with elevated oxidative stress, which, in turn, triggers increased NO expression [78,79]. Increased reactive oxygen species and decreased antioxidant defenses can influence the pathophysiology of inflammation, fibrosis, reperfusion injury, atherosclerosis, Alzheimer’s disease, and chronic obstructive pulmonary disease [80,81]. Thus, it is suggested that the isolated consumption of black cricket in this study was associated with oxidative damage in the animals’ liver, while its combination with soy flour proved to be beneficial.

The limitations of this study should be acknowledged when interpreting the results. First, the use of rodents as an experimental model is a well-established method for assessing iron bioavailability, using a depletion–repletion model; however, this methodology is not used in human research due to ethical and practical constraints. Additionally, the lack of intestinal microbiota analysis limits our understanding of the diets’ effects on microbial balance and its potential role in nutrient absorption. Despite these limitations, the results of this study are promising, suggesting that the combination of *Gryllus assimilis* powder with soy flour may benefit individuals and populations vulnerable to iron deficiency. Future research could explore the impact of these findings in human populations through observational studies or controlled supplementation trials, particularly in groups at risk of iron deficiency.

## 5. Conclusions

In summary, the results of this study indicate that *Gryllus assimilis* powder, when combined with soy flour, is an effective source of bioavailable iron, supporting the maintenance of hemoglobin levels. This combination also improved intestinal health by increasing acetic acid and fecal moisture and promoted oxidative balance, with higher superoxide dismutase activity compared to the isolated powder, which showed more peroxidation products.

## Figures and Tables

**Table 1:** Proximate composition of soybean flour and *Gryllus assimilis* powder on a dry basis.

Compounds	*Gryllus assimilis*	Soy Flour
Moisture (g/100 g)	3.68 ± 0.01	5.92 ± 0.23
Ash (g/100 g)	4.04 ± 0.16	5.02 ± 0.05
Lipids (g/100 g)	18.28 ± 1.01	22.55 ± 0.59
Proteins (g/100 g)	64.75 ± 0.79	43.90 ± 1.63
Total dietary fiber (g/100 g)	9.04 ± 0.01	13.35 ± 0.48
Carbohydrates (g/100 g)	0.21 ± 0.01	9.26 ± 2.75
Iron (mg/100 g)	23.72 ± 1.31	7.94 ± 0.69

Data presented as the mean ± standard deviation.

**Table 2:** Total consumption, body weight gain, iron intake, iron utilization, and indices for assessing iron bioavailability.

Variables	SF	GP + FS	GP	FS
Total intake (g)	338.41 ± 15.98 ^a^	348.82 ± 17.91 ^a^	343.61 ± 32.30 ^a^	342.28 ± 18.17 ^a^
Weight gain (g)	30.42 ± 18.85 ^a^	32.81 ± 20.41 ^a^	30.04 ± 19.13 ^a^	20.33 ± 16.89 ^a^
FER	0.09 ± 0.05 ^a^	0.10 ± 0.05 ^a^	0.10 ± 0.04 ^a^	0.8 ± 0.04 ^a^
Iron intake (g)	7.39 ± 0.35 ^ab^	8.02 ± 0.41 ^a^	7.90 ± 0.74 ^ab^	7.29 ± 0.39 ^b^
HRE %	41.37 ± 10.86 ^a^	41.13 ± 10.86 ^a^	36.40 ± 6.26 ^a^	40.82 ± 8.03 ^a^
RBV-HRE	1.00 ± 0.15 ^a^	0.99 ± 0.18 ^a^	0.88 ± 0.12 ^a^	0.98 ± 0.11 ^a^
Iron utilization	0.90 ± 0.14 ^ab^	0.94 ± 0.15 ^a^	0.83 ± 0.11 ^b^	0.86 ± 0.10 ^ab^

Data are presented as the mean ± standard deviation. N = 8 animals/group. SF: Ferrous Sulfate; GP + FS: *Gryllus assimilis* powder associated with soybean flour (15:85); GP: *Gryllus assimilis* Powder; FS: Soybean Flour. Means followed by different lowercase letters in the same line indicate a significant difference, according to a Tukey’s test at a 5% probability.

**Table 3:** Hemoglobin values, hemoglobin gain, and serum ferritin and transferrin levels in the repletion phase.

Variables	SF	GP + FS	GP	FS
Initial hemoglobin (g/dL)	7.52 ± 1.51 ^a^	7.51 ± 1.47 ^a^	7.57 ± 1.47 ^a^	7.67 ± 1.38 ^a^
Final hemoglobin (g/dL)	14.46 ± 1.99 ^a^	14.49 ± 1.25 ^a^	12.05 ± 1.82 ^b^	14.37 ± 1.01 ^a^
Hemoglobin gain (g/dL)	7.53 ± 2.00 ^a^	6.98 ± 1.08 ^a^	4.49 ± 2.00 ^b^	6.70 ± 1.38 ^ab^
Transferrin (mg/dL)	163.40 ± 8.26 ^b^	178.03 ± 4.90 ^a^	185.91 ± 8.19 ^a^	160.22 ± 7.44 ^b^
Ferritin (ng/dL)	5.18 ± 1.01 ^ab^	4.56 ± 0.97 ^b^	6.41 ± 1.10 ^a^	4.64 ± 0.84 ^ab^

Data are presented as the mean ± standard deviation. N = 8 animals/group. SF: Ferrous Sulfate; GP + FS: *Gryllus assimilis* powder associated with soybean flour (15:85); GP: *Gryllus assimilis* Powder; FS: Soybean Flour. Means followed by different lowercase letters in the same line indicate a significant difference, according to a Tukey’s test at a 5% probability.

**Table 4:** Analyses of short-chain fatty acids, moisture, and pH of cecal feces.

Variables	SF	GP + FS	GP	FS
Acetic acid (mmol/L)	7.58 ± 0.92 ^d^	10.02 ± 0.76 ^c^	11.59 ± 0.97 ^b^	13.95 ± 1.80 ^a^
Propionic acid (mmol/L)	3.60 ± 0.53 ^b^	3.86 ± 0.64 ^b^	4.27 ± 0.47 ^ab^	4.78 ± 0.21 ^a^
Butyric acid (mmol/L)	0.99 ± 0.12 ^a^	1.17 ± 0.32 ^a^	1.31 ± 0.29 ^a^	1.15 ± 0.27 ^a^
Total SCFA	12.56 ± 1.84	15.87 ± 0.86	17.24 ± 1.74	19.33 ± 1.63
Fecal moisture (%)	12.32 ± 1.37 ^b^	21.29 ± 3.80 ^a^	16.08 ± 2.53 ^b^	18.72 ± 1.30 ^ab^
Cecal stool pH	8.25 ± 0.69 ^a^	7.96 ± 0.31 ^a^	7.74 ± 0.36 ^a^	8.03 ± 0.55 ^a^
Stool color	3.00 ± 0.00 ^a^	2.00 ± 0.00 ^a^	2.00 ± 0.00 ^a^	1.00 ± 0.00 ^a^
Stool consistency	2.00 ± 0.00 ^a^	1.00 ± 0.00 ^a^	2.00 ± 0.00 ^a^	1.00 ± 0.00 ^a^

Data are presented as the mean ± standard deviation. N = 8 animals/group. SF: Ferrous Sulfate; GP + FS: *Gryllus assimilis* powder associated with soybean flour (15:85); GP: *Gryllus assimilis* Powder; FS: Soybean Flour. Means followed by different lowercase letters in the same line indicate a significant difference, according to a Tukey’s test at a 5% probability.

**Table 5:** Effect of experimental diets on duodenal histomorphometry.

Variables	SF	GP + FS	GP	FS
Mucosal thickness (µm)	600.64 ± 78.48 ^b^	725.26 ± 79.41 ^ab^	798.81 ± 78.53 ^a^	796.78 ± 46.29 ^a^
Submucosal thickness (µm)	47.14 ± 7.68 ^a^	46.24 ± 3.88 ^a^	48.84 ± 3.20 ^a^	45.23 ± 10.31 ^a^
Goblet cell diameter (µm)	16.86 ± 0.94 ^b^	17.95 ± 0.97 ^b^	19.21 ± 0.92 ^a^	16.78 ± 0.96 ^b^

Data are presented as the mean ± standard deviation. N = 8 animals/group. SF: Ferrous Sulfate; GP + FS: *Gryllus assimilis* powder associated with soybean flour (15:85); GP: *Gryllus assimilis* Powder; FS: Soybean Flour. Means followed by different lowercase letters in the same line indicate a significant difference, according to a Tukey’s test at a 5% probability.

## Data Availability

The data will be shared upon reasonable request to the corresponding author due to ethical restrictions related to the use of animals in research.
